# A37 FECAL CALPROTECTIN IN A PEDIATRIC, POPULATION-BASED STUDY: UTILITY IN DIAGNOSIS AND INFLAMMATORY BOWEL DISEASE MONITORING

**DOI:** 10.1093/jcag/gwac036.037

**Published:** 2023-03-07

**Authors:** M Smyth, S Lawrence, V Barakauskas, H Vallance, K Jacobson

**Affiliations:** 1 Pediatric GI; 2 Pathology and Laboratory Medicine; 3 University of British Columbia, Vancouver, Canada

## Abstract

**Background:**

Fecal calprotectin (FC) is a sensitive marker of intestinal inflammation, and is used to both discriminate inflammatory bowel disease (IBD) from non-IBD patients and to monitor patients with IBD. It is unclear whether normal values established in adult patients are applicable in pediatrics.

**Purpose:**

To evaluate FC’s ability to differentiate IBD from non-IBD pediatric patients, and to understand factors influencing FC in pediatric IBD(pIBD).

**Method:**

Stool FC samples collected on all patients<19 years of age in British Columbia(BC) from May 2020 to August 2022 were run using a Buhlmann ELISA at BC Children’s Hospital(BCCH). The BCCH GI database identified patients with IBD. FC’s ordered by adult IBD providers and patients awaiting endoscopy were excluded. The remaining samples were analysed as non-IBD. The sensitivity(Sn), negative predictive value(NPV) and false positive(FP) of FC were evaluated; comparisons were made using the Wilcoxon rank-sum test and chi-squared.

**Result(s):**

3506 FC samples met inclusion criteria: 1853 IBD and 1653 non-IBD. 221 IBD samples were from prior to diagnosis, with median (IQR) FC 2615ug/g(1090-4183); median FC for non-IBD patients was 54ug/g(24-122). Using the Buhlmann "normal" cutoff of 80ug/g, the Sn was 0.991 (NPV 0.998) with a FP rate of 37%. Young patients were more likely to have FP's: <6yo's (n=305) had a FP rate of 42% vs 36% in those >6yo (n=1348)(p=0.035). With a FC cutoff of 160ug/g, Sn was 0.973 (NPV 0.996), with a FP rate of 20% (24% <6yo vs 19% >6yo, p=0.025). At a threshold of 250ug/g, Sn was 0.959 (NPV 0.994) with a FP rate of 13% (18% <6yo vs 12% >6yo, p=0.01). For patients <2yo, all 4 new IBD diagnosis had FC>1900ug/g. In the non-IBD population (n=69 samples <2yo), the FP rate was 52%, 30%, and 22% using a threshold of 80, 160, and 250ug/g, respectively.

Evaluating FC as a disease-monitoring tool in IBD (667 patients, 1632 samples) found that at 6, 12, 18, and 24+ months post diagnosis, FC decreased from 750(159-1883), 505(110-1566), 351(87-1379), to 308ug/g(85-1129), respectively. Similarly, the proportion of FC’s <250ug/g increased from 4.1% at diagnosis to 30%, 39.7%, 41.5%, 47% during the follow up period.

Patients with UC/IBD-U had higher FC’s, and were less likely to achieve FC<250ug/g. By 12 months post diagnosis, median FC of CD patients was 347ug/g(96-1150) and UC/IBD-U was 745ug/g(191-2017)(p=0.036), and at 18 months, CD 273ug/g(61-902) vs UC/IBD-U 932ug/g(144-2229)(p<0.001). At 2+yrs, median FC for CD patients was 259ug/g(76-1038) vs 387ug/g(108-1577) for UC/IC (p=0.017).

Patients cared for by an IBD specialist had better FC outcomes vs those managed by non-IBD GIs: median FC at 12 months was 246ug/g(n=79) vs 677ug/g(n=153)(p=0.002), with 51% vs 34% achieving FC<250(p=0.014). Similarly, at 2+yrs post diagnosis, median FC was 243ug/g(n=437) vs 356ug/g(n=407)(p=0.02).

**Conclusion(s):**

Higher FC thresholds are likely required in younger populations compared to established adult cutoffs. In this pIBD cohort, <50% achieve FC levels <250ug/g.

**Please acknowledge all funding agencies by checking the applicable boxes below:**

None

**Disclosure of Interest:**

None Declared

